# Modulatory effects of T-cell immunosuppression on pigeon protozoal encephalitis induced by *Sarcocystis calchasi*

**DOI:** 10.1038/s41598-025-17151-6

**Published:** 2025-12-21

**Authors:** Saskia Nemitz, Achim D. Gruber, Tobias Britzke, Anne Voss, Ronja Rahner, Kathrin Büttner, Andreas R. Schaubmar, Michael Lierz, Kristina Maier-Sam

**Affiliations:** 1 Clinic for Birds, Reptiles, Amphibians & Fish, Justus Liebig University, Giessen, Germany; 2 Institute of Veterinary Pathology, Freie Universität Berlin, Berlin, Germany; 3 Unit for Biomathematics and Data Processing, Justus Liebig University, Giessen, Germany

**Keywords:** Immunology, Diseases, Neurology

## Abstract

*Sarcocystis calchasi* is the causative agent of Pigeon Protozoal Encephalitis, a neurological disease in pigeons. The biphasic disease is characterized by neurological signs in the chronic phase. Parasite stages are generally not associated with inflammatory brain lesions and the parasite has been suggested to modulate the host’s immune system. To test this hypothesis, pigeons experimentally infected with *S. calchasi* were T-cell immunosuppressed beginning from 14 days post infection (dpi) until the end of the experiment (59/60 dpi) and compared with immunocompetent animals. When scored histologically (sum encephalitis score consisting of lympho-histiocytic perivascular cuffs, lymphocytic encephalitis and gliosis), encephalitis was markedly less pronounced in immunosuppressed pigeons than in immunocompetent animals (6.8 ± 4.4 s.d. versus 11.2 ± 3.0 s.d.). Thus, the alleviation of the disease by immunosuppression supports the hypothesis of an immune-mediated mechanism rather than direct damage by the pathogen. Results from a second infection trial, where the effect of immunosuppression only during early (12–20 dpi) or late phase (30 dpi – end of experiment) was compared, did not show significant differences between both groups and suggest that immunomodulation is triggered during the early stage of parasite development by sporozoites and/or more likely merozoites.

## Introduction

*Sarcocystis (S.) calchasi* is an apicomplexan parasite with an obligate two-host life cycle^1^. Birds of the order Accipitriformes have been identified as definitive hosts^2,3^, with a wide range of susceptible intermediate hosts, including birds of the orders Columbiformes, Psittaciformes, Piciformes, Suliformes and Galliformes^4–13^. As usual in the life cycle of *Sarcocystis* species, intermediate hosts, e.g. domestic pigeons (*Columba livia* f. *domestica*), are infected by oral ingestion of food or water contaminated with sporocysts that have been shed via the faeces of definitive host species, such as northern goshawks (*Accipiter gentilis*). After excystation in the intestine, sporozoites of *S. calchasi* migrate to the liver where merozoites replicate by schizogony at approximately 10 days post infection (dpi), causing necrotizing hepatitis with lymphocyte infiltration^14–17^. This asexual reproductive phase is followed by invasion of striated musculature and the formation of characteristic muscular cysts. Immature sarcocysts in the skeletal muscle and myocardium can be seen from 20 dpi onwards, characterized by round, pale basophilic metrocytes. At the same time a mild lymphocytic encephalitis begins to develop. From 30 dpi onwards, mainly mature sarcocysts with strong basophilic, elongated cystozoites are present in striated muscles, alongside a few immature cysts. Severity of encephalitis increases from 47 dpi onwards resulting in neurological signs^17,18^. The diseased intermediate host with muscle sarcocysts as infective stage for the definitive host can thus be more easily predated, completing the parasite´s life cycle.

The neurological disease caused by *S. calchasi* was first described in pigeons and thus named Pigeon Protozoal Encephalitis (PPE)^19^. It is characterized by a dose-dependent biphasic course: During the acute phase around 10 dpi pigeons infected with moderate doses (2 × 10^2^ – 10^4^, depending on strain virulence) show mainly apathy and polyuria, while neurological signs such as ataxia, equilibrium disorders and torticollis define the chronic phase, starting around 47 dpi. Lower infectious doses result in a clinically obscure acute phase followed by a manifest chronic phase, whereas overtly high doses may result in acute death of the host during the first phase.

Histologically, PPE is characterized by granulomatous and necrotizing encephalitis from 20 dpi onwards, but parasitic stages are only rarely found in the brain and are usually not associated with the lesions^17,18,20^. The underlying pathomechanism of the brain lesions and the subsequent neurological signs has not yet been identified. Moreover, there is no apparent correlation between the severity of the encephalitis and the number of sarcocysts. It has therefore been suggested that *S. calchasi* may be capable of modifying the intermediate host’s immune system^17^. Specifically, a down-regulation of the Th1 immune response has been observed during the acute phase with significant lower levels of Th1-associated cytokines, such as IFN-γ, interleukin (IL)-12 and IL-18, possibly indicating an immune evasion strategy of the parasite. In contrast, the Th1 immune response is upregulated during the chronic phase with increased levels of IFN-γ and tumor necrosis factor (TNF) α-related cytokines^20^. It has therefore been debated whether encephalitis is caused by only early and temporary neuroinvasion of schizonts (hit-and-run mechanisms) or by immunomodulation, possibly a delayed type IV hypersensitivity reaction or autoreactive lymphocytes or antibodies induced by merozoites or immature cysts^17^. The upregulation of Th1-associated cytokines during the chronic phase of PPE seems to support the second hypothesis.

Here, we aimed to provide more experimental evidence to specify the potential impact of immunomodulation on the progression of encephalitis. To this end, we assessed the effects of selective T-cell immunosuppression by ciclosporin (CsA) on the course of PPE in an experimental infection setup. In a second experiment, CsA was administered either during the early (12–20 dpi, before the development of sarcocysts) or late phase (30 dpi – end of experiment, during the presence of sarcocysts) after infection to more precisely identify the time point and parasite developmental stages likely to be involved in the proposed immunomodulation.

## Materials and methods

**Fig. 1:**
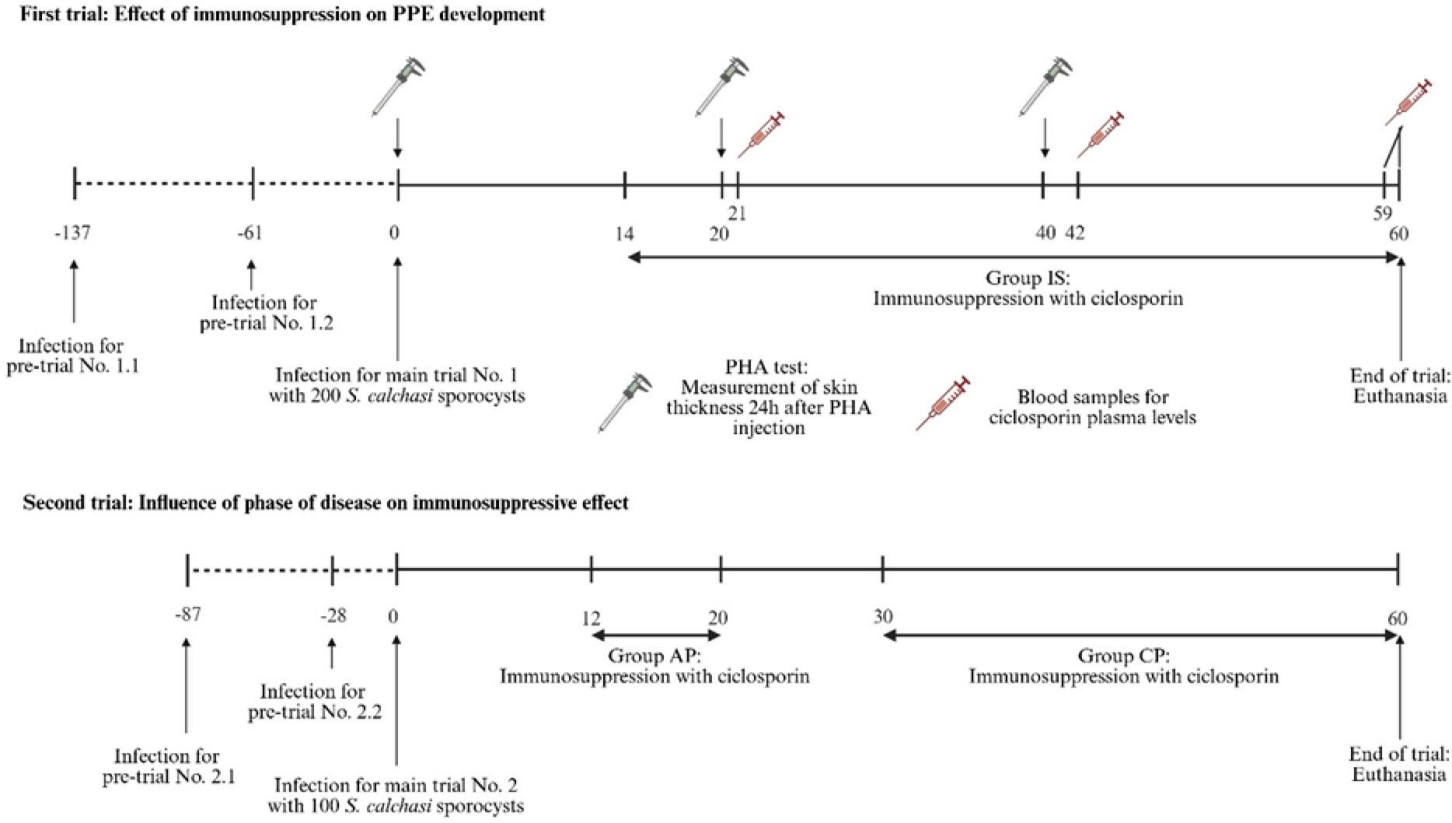
Experimental design of the trials. (Created in BioRender. Maier-Sam, K. (2025), https://BioRender.com/6q9iktg)

Domestic pigeons were experimentally infected with *S. calchasi* sporocysts in a two-stage setup (Fig. 1). In the first step, pigeons were T-cell immunosuppressed with ciclosporin from 14 dpi onwards and compared to immunocompetent infected pigeons using clinical and histopathological scores. In the second setup, pigeons immunosuppressed during the early schizogonic phase of PPE were compared to pigeons treated with CsA only during the late neurological phase. Prior to the main experiments, pre-trials were conducted to determine the optimal infectious dose with regards to the clinical course of immunocompromised pigeons. Pigeons were euthanized by exsanguination under general anaesthesia with isoflurane at the end of the experiments or when reaching humane endpoints (severe general or neurological signs or moderate general or neurological signs that lasted for more than 48 h).

All experiments involving live animals were ethically reviewed and approved by the Regierungspräsidium Giessen (approval numbers: V 54 − 19 c 20 15 h 01, GI 18/5, Nr. G 32/2018 and V 54 − 19 c 20 15 h 01, GI 18/9, Nr. G 91/2022). All methods were conducted in accordance with relevant guidelines and regulations and reported in accordance with ARRIVE (Animal Research: Reporting of In Vivo Experiments) guidelines.

### Pigeons

Adult male and female racing pigeons were acquired from a private pigeon breeder and originated from a controlled breeding flock. The pigeon breeder gave his informed consent for using the pigeons in this study. They were housed and reared in an enclosed aviary without contact to free-ranging birds or their excreta. Prior to the trials, infection with *Salmonella* spp. or *S. calchasi* was excluded by repeated cultivation of pooled faecal samples and PCR examination (see below) of a biopsy of the pectoral muscle, respectively. Crop swabs were repeatedly tested to exclude an infection with *Trichomonas* spp. and blood was tested by hemagglutination inhibition assay to exclude the presence of specific antibodies against *Avian Orthoavulavirus* 1. Faecal samples were furthermore examined for the absence of endoparasites. The pigeons were randomly assigned to the different experimental groups using the “rand”-function in Excel (Microsoft, Redmond, USA). Before and during the trials, the pigeons were housed as a single flock in an indoor aviary and checked daily for clinical signs.

### Source of *S. calchasi* sporocysts

The sporocysts used in this study belonged to the Giessen16 strain. The sporocysts were originally obtained from the intestine of a naturally *S. calchasi* infected northern goshawk, purified as previously described^21^ and stored in Hank’s Balanced Salt Solution (HBSS) supplemented with penicillin/streptomycin and amphotericin B at 4 °C. To maintain their viability and infectivity, they were passaged every two years through their natural hosts (Regierungspräsidium Giessen, approval number: GI 18/5 A7/2018). For this, a northern goshawk was fed with the pectoral muscle of an experimentally infected domestic pigeon (40 dpi). Fourteen days after the infection of the northern goshawk, it was euthanized, mucosal scrapings from the intestine were collected and sporocysts were purified by trypsin ingestion and filtering^21^.

### Experimental infection

Pigeons were infected with *S. calchasi* sporocysts belonging to the Giessen16 strain. Sporocysts were counted using a Neubauer counting chamber and suspended in tap water. The pigeons then received the suspension with the appropriate sporocyst dose (see above) orally. Pigeons in the control groups received tap water instead. All pigeons were kept separately for 72 h to avoid passive transmission of potentially passaged sporocysts between the pigeons before being transferred to the indoor aviary.

### Pre-trials

Previous studies have shown that storage of *S. calchasi* sporocysts may be associated with an increase in virulence^17^. Therefore, a pre-trial was performed prior to each main trial to determine the appropriate dose required to induce the biphasic disease typical of PPE. Prior to the first main trial, we determined an infectious dose with no or only mild acute phase as this would ensure successful infection and a later development of the chronic phase^17^. To this end, two groups of three pigeons each were infected with 200 or 400 sporocysts of *S. calchasi* and euthanized at 15 dpi as the acute phase is usually clinically evident at 10 dpi (pre-trial No. 1.1). A second pre-trial was designed to determine the effect of CsA treatment on the severity of the acute phase of PPE (pre-trial No. 1.2). For this purpose, two groups of six infected pigeons each (dose from pre-trial No. 1.1) received CsA beginning on the day before infection (-1 dpi) or at 8 dpi and all were euthanized at 15 dpi or at the onset of severe clinical signs consistent with the acute phase of PPE. As the second main trial was conducted 15 months after the first main trial and the parasite had to be passaged through its natural hosts in between to maintain its viability, the pre-trial for dose determination was repeated (pre-trial No. 2.1 with 400 sporocysts per pigeon and 2.2 with 50/100/200 sporocysts per pigeon).

### Main trials

Based on the results of the pre-trials, in the first main trial (main trial No. 1) 26 pigeons were orally infected with 200 sporocysts of *S. calchasi* each and divided into two groups, one group (*n* = 13) receiving CsA treatment (IS) and the other (*n* = 13) serving as an immunocompetent reference group (IC). Two further groups of three uninfected pigeons each served as controls for the effects of CsA treatment (C1) alone and environmental factors (C2). Groups IS and C1 received CsA from 14 dpi onwards until the end of the experiment. Groups IC and C2 received no treatment. All pigeons were euthanized at 59 or 60 dpi or at the onset of severe clinical signs during the chronic phase of PPE. In the second main trial (main trial No. 2), 32 pigeons were infected with 100 sporocysts according to the results of the repeated pre-trial and divided into two groups that received CsA treatment either during the acute phase of PPE (*n* = 16) (AP) or during the chronic phase (*n* = 16) (CP). Three uninfected and untreated pigeons served as a control group for environmental factors (C3). The infected groups were treated with CsA, group AP daily from 12 to 20 dpi (before the formation of immature sarcocysts with presumably merozoites as the main parasitic stage), group CP daily from 30 dpi onwards (when mature sarcocysts are present in the muscles) until the end of the experiment (dpi 60).

### Immunosuppression

Sporimune (50 mg/ml ciclosporin, Dechra Veterinary Products, Aulendorf, Germany) was used to reduce T-lymphocyte activity. It was administered orally at a dose of 30 mg/kg once daily for the period described above for each group^22^. Blood samples were collected from the basilical vein at 21, 42 and 59/60 dpi in main trial No. 1 and analyzed via mass spectrometry by Laboklin (Bad Kissingen, Germany) to monitor CsA plasma levels. In addition, samples from the immunocompetent groups IC and C2 were collected at the end of the experiment to assess the cut-off value.

### Phytohemagglutination test

To verify the effect of CsA on the T-cell immune response, at -1, 19 and 39 dpi during the first main trial four randomly selected pigeons from the immunosuppressed groups were injected with 0.1 ml phytohemagglutinin dissolved in PBS (1 mg/ml; phytohemagglutinin PHA-P, Sigma-Aldrich, St. Louis, USA) into the wing span skin of the right wing and 0.1 ml phosphate-buffered saline (PBS) into the left wing as a control. Injection of phytohemagglutinin (PHA) results in a measurable increase in skin thickness in immunocompetent pigeons because it induces an accumulation of T-lymphocytes in the perivascular tissue^23^. Immunocompromised animals typically develop a lower increase in thickness when compared to the PBS injection site^24^. Here, the T-cell mediated immune response was estimated as the increase in skin thickness on the PHA side minus the increase on the PBS side after 24 h.

### Clinical examination

After the onset of the chronic phase of PPE, the behaviour as well as general clinical and neurological signs were scored daily with scores from 0 = no signs to 3 = severe signs (Table 1). At 52, 54, 56 and 58 dpi (main trial No. 1) and 54 and 56 dpi (main trial No. 2) respectively, scoring was performed in a blinded fashion with the examiner being unaware of the group assignment. Pigeons that reached a score 3 or showed score 2 for more than 48 h were euthanized for animal welfare reasons.

**Table 1:** Scoring system for clinical scores.

	Score	Parameters
General signs	0	Smooth, clean & glossy plumage, shiny eyes, clean body orifices
	1	Partially dull, slightly fluffed plumage and/or cloudy, partially closed eyes
	2	Partially dull, slightly fluffed plumage, eye and nose discharge, sunken and cloudy, partially closed eyes and/or sticky or moist body orifices
	3	Completely dull, fluffed plumage, retracted head, arched back, eyes closed, cyanosis and/or breathing noises
Behaviour	0	Attentive, active, social contacts, normal movement
	1	Reduced reactions, reduced movement and/or limited or excessive activity
	2	Partial separation from the group, reduced movement and/or evident pain during movement
	3	Apathy, no reaction or aggression during handling, isolation and/or pronounced stereotypies or hyperkinetics
Neurological signs	0	No neurological signs
	1	Mild ataxia, paresis, equilibrium disorders and/or torticollis
	2	Moderate ataxia, paresis, equilibrium disorders and/or torticollis
	3	Severe ataxia, paralysis, equilibrium disorders and/or torticollis

### Pathology and histopathology

Complete necropsy was performed on all pigeons and the following organs were collected for standard histopathology: brain, spinal cord from the cervical spine, skeletal muscle (pectoral and femoral muscles, three distinct locations each), bone marrow, heart, lung, liver, spleen, kidney, crop, proventriculus, ventriculus and small and large intestine. Only liver and pectoral muscle tissue samples were collected from the pigeons in main trial No. 2 that were euthanized in the acute phase of PPE. Mucosal scrapings from various locations in the small and large intestine were examined for endoparasites and samples of heart blood, liver and lung were cultured on Columbia sheep blood agar at 37 °C for 48 h to monitor for bacterial infection. Tissue samples from all organs were fixed in 10% buffered formalin. Liver samples from the pigeons in the acute phase as well as samples from skeletal muscles and brain from the pigeons in the chronic phase were collected for PCR analysis to detect *S. calchasi* genomic sequences (see below).

**Fig. 2:**
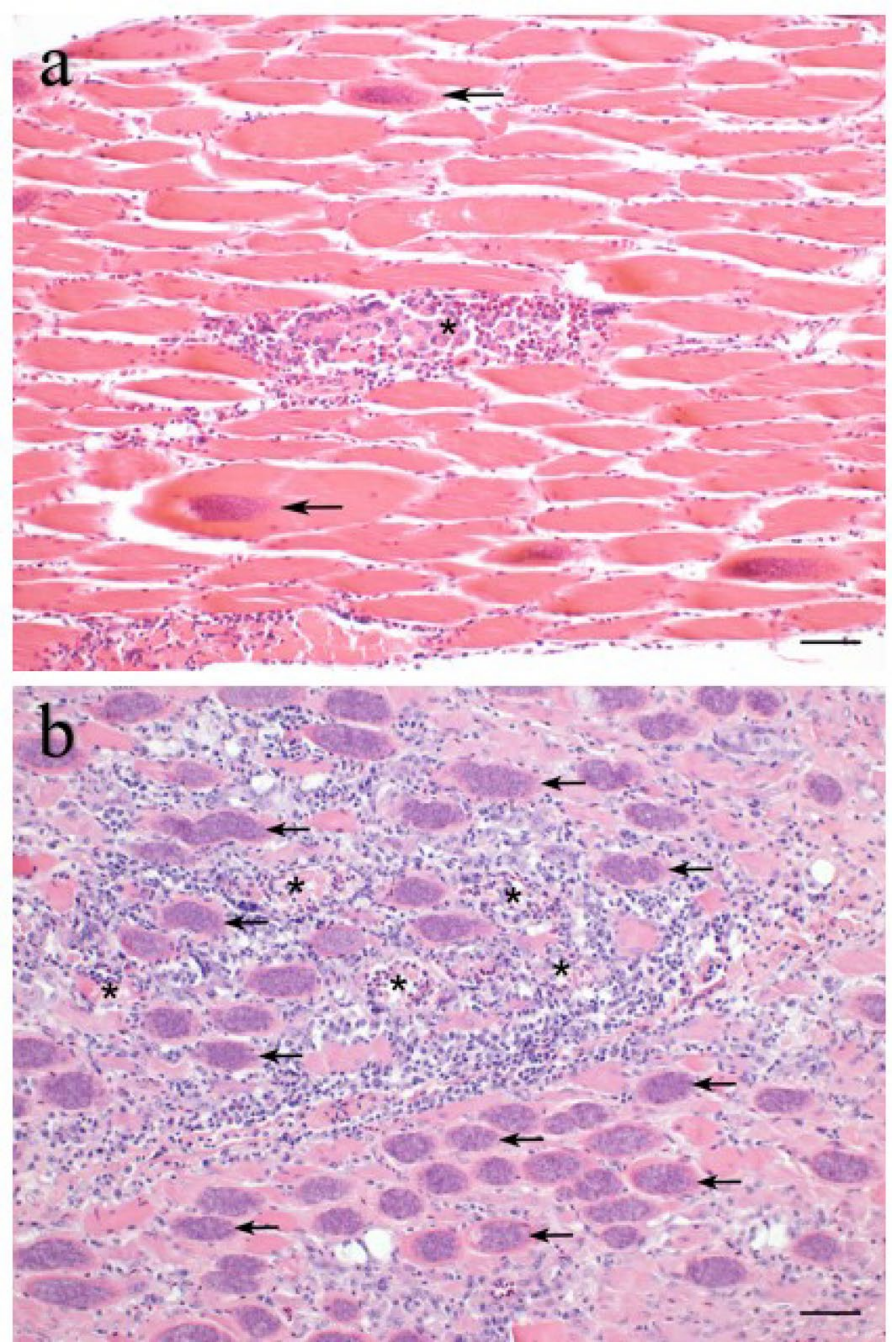
Sarcocysts. Representative histomicrographs of sarcocysts in the pectoral muscles with varying degrees of lymphohistiocytic inflammation. Different degrees of infection were sum scored from score 1 (**a**) to score 5 (**b**). *S. calchasi* cysts within skeletal myocytes (arrows) and myonecrosis with lymphohistiocytic and eosinophilic inflammation (asterisk). Hematoxylin and eosin stain, scale bars: 100 μm.

**Fig. 3:**
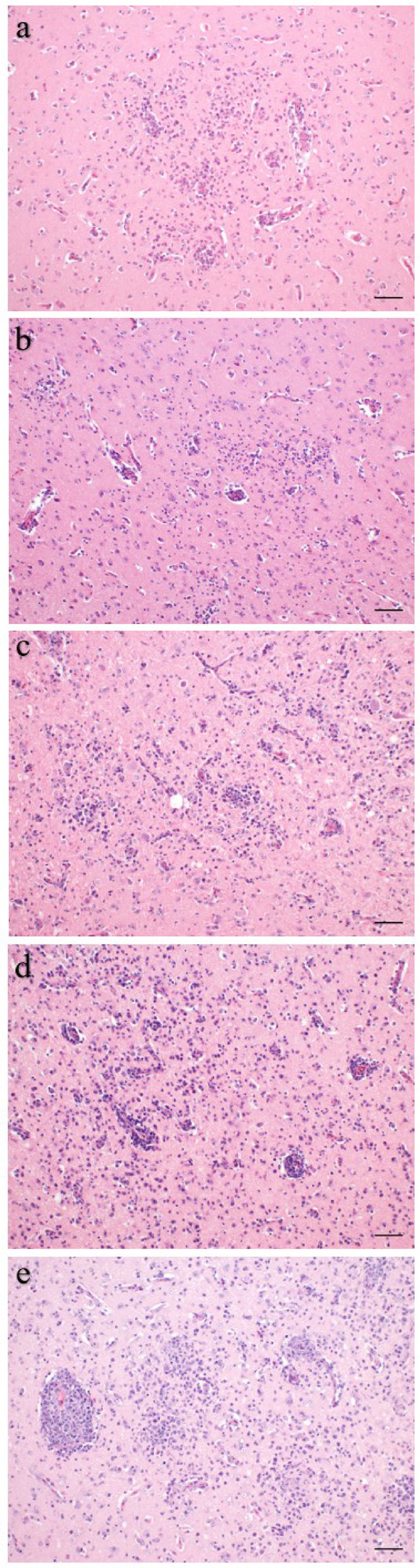
Encephalitis. Representative histomicrographs of encephalitis. Different severities were sum scored as score 1 (**a**), 2 (**b**), 3 (**c**) 4 (**d**), or 5 (**e**) depending on the degree of lymphohistiocytic infiltration, perivascular cuffing and gliosis. Hematoxylin and eosin stain, scale bars: 100 μm.

Formalin-fixed organ samples were routinely embedded in paraffin, sectioned at 3 μm thickness and stained with hematoxylin and eosin. A scoring system was established for histopathological analysis of the brain, skeletal muscle, heart and liver. Samples were scored in a blinded fashion with the examiner being unaware of the group assignments. The histopathological scoring was performed by a single examiner to avoid inter-observer-variability after training and harmonization of scoring results with a second examiner on the same lesions. The right brain hemisphere was cut into 5 evenly spaced frontal sections and representative sections of the cerebrum, hippocampus and cerebellum were scored for the overall degree of inflammation, characterized by lympho-histiocytic encephalitis, perivascular immune cell cuffs, gliosis and necrosis. Immune cell infiltration consisting of lymphocytes, macrophages and heterophils as well as necrosis and parasite infection were scored in slides of skeletal muscle, heart, liver and spleen. In addition, liver samples were scored for the extent of multifocal infiltration with lymphofollicular aggregates. For each parameter the sample with the most severe lesion was selected and assigned a score of 5. All other samples were scored according to their relative degree of severity as compared to the score 5 samples (Table 2; Figs. 2 and 3). Kidney, proventriculus, ventriculus, intestine and bone marrow were also examined histologically in terms of descriptive pathology to identify possible other related or unrelated tissue lesions. Microscopic images were taken on an Olympus BX41 microscope (Olympus Corporations, Tokio, Japan) with an Olympus DP80 camera with Olympus cellSens standard software (Version 1.18, Olympus Corporations, Tokio, Japan). Automatic white balance and sharpening were applied uniformly to all images using Adobe Photoshop CS6 Version: 13.0 × 64 (Adobe Systems Inc., San José, USA).

**Table 2:** Scoring system for histological scores.

Score	Estimated severity in percent of the most strongly affected sample
1	0–20%
2	20%-40%
3	40%-60%
4	60%-80%
5	80%-100%

For each parameter, the sample with the most severe lesion was selected and assigned a score of 5. All other samples were scored according to their relative degree of severity compared to the score 5 sample.

### Statistical evaluation

For encephalitis score as primary outcome measure, the sum of all three histopathologically assessed encephalitis criteria (lymphohistiocytic encephalitis, perivascular immune cell cuffs and gliosis) was calculated and compared between the groups. All successfully infected pigeons that were euthanized during the chronic phase of PPE were included for analysis. Statistical analysis was performed with SAS 9.4 (SAS Institute Inc., Cary, NC, USA), using a one-tailed Mann-Whitney-U-Test for the encephalitis and sarcocyst scores in main trial No. 1 to compare the scores from groups IS (*n* = 5) and IC (*n* = 6), and a two-tailed Mann-Whitney-U-Test for the encephalitis score in main trial No. 2, to compare the scores of groups AP (*n* = 6) and CP (*n* = 5).

### Immunohistochemistry

Immunohistochemistry (IHC) was performed as previously published^25^. Briefly, formalin-fixed paraffin-embedded tissue sections were deparaffinised in xylene and rehydrated through graded ethanols. Slides were incubated in 0.5% H_2_O_2_ for 20 min and antigen retrieval was achieved by 0.1% protease pretreatment for 10 min at 37 °C. Tissues were blocked with 20% normal goat serum and 10% Roti-Immunoblock (Roth, Karlsruhe, Germany) for 30 min and incubated with a cross-reactive rabbit anti-*S. neurona* antibody (dilution: 1:2,500; kindly provided by Dan K. Howe, Maxwell H. Gluck Equine Research Center Veterinary Science, University of Kentucky, USA) overnight at 4 °C. After washing, a goat anti-rabbit polymer (Goat-Anti-Rabbit IgG, Vector Laboratories Inc., Burlingame, USA) was applied for 30 min. Avidin–biotin–peroxidase complex (ABC) solution (Vectastain Elite ABC Kit; Vector Laboratories) and diaminobenzidine (DAB) were used for colour development. Sections were counterstained with Mayer’s hematoxylin, rehydrated and cover slipped. To control for proper reactivity of the anti-S. neurona antibody and general protocol efficacy, pectoralis muscle samples of experimentally S. calchasi-infected pigeons containing typical parasitic cysts were included as well as brain tissues from pigeons that had been previously experimentally infected with S. calchasi^17^. Irrelevant rabbit immunoglobulins (Bio-Genex, Fremont, USA) were used to control for specificity of antibody-binding. Furthermore, brains and pectoralis muscle of uninfected pigeons that tested negative for S. calchasi DNA by PCR were used to further control for specificity of the antibody. The immunohistochemistry images were achieved as previously described for histological images.

### Semi-nested polymerase chain reaction

Skeletal muscle and brain samples from pigeons euthanized in the chronic phase and liver samples of the pigeons euthanized or died in the acute phase of PPE were analyzed by PCR for the detection of *S. calchasi*-DNA. DNA was extracted from the samples using the DNeasy Blood & Tissue Kit (Qiagen, Hilden, Germany) according to the instructions and then measured (NanoDrop 2000cs spectrophotometer; Thermo Fisher Scientific, Wilmington, DE, USA) and standardized to 5 ng/µl. DNA extracted from *S. calchasi* sporocysts, added as a serial tenfold dilution, served as positive control and RNase-free water was used as negative control. Semi-nested PCR targeting the internal transcribed spacer (ITS1) region of *S. calchasi* was performed as previously published, using primers SCa1 (5’- CTC CTT GCT CGA GAA TGA ACA TGA G - 3’) and SCa2 (5’-GAT CAT CTT TTC GAC GAC AAT ATC G - 3’) for the first amplification and primers SCa1 and SNCa3 (5’ – TCC AGA GAA GAT CCC CTG GCT AC - 3’) for the second amplification^17^.

## Results

### Determination of infectious dose

In pre-trial No. 1.1 both infectious doses of 200 and 400 sporocysts with an infection rate of 100% did not induce any clinical signs during the acute phase of PPE. For main trial No. 1, 200 sporocysts per pigeon were chosen as the infectious dose. In pre-trial No. 1.2 an increased lethality was observed in both groups (2 out of 6 pigeons per group), indicating a severe aggravation of the clinical outcome during the acute phase due to immunosuppression. Therefore, the start of the immunosuppressive intervention in main trial No. 1 was scheduled at 14 dpi after the acute phase. In pre-trial No. 2.1 400 sporocysts per pigeon resulted in the death of all pigeons at 10 dpi indicating a severe increase in virulence after storage or passage. In pre-trial No. 2.2 all pigeons developed mild clinical signs such as fluffing and reduced activity with no difference between the pigeons dosed with 50, 100 or 200 sporocysts. Therefore, the infectious dose for main trial No. 2 was chosen at 100 sporocysts per pigeon to minimize the risk of mortality during the acute phase but maximize the chance of successful infection. For main trial No. 2, immunosuppression of group AP was initiated at 12 dpi, when most of the clinical signs of the acute phase are subsided, to start as early as possible after infection.

### Evaluation of immunosuppression

**Fig. 4:**
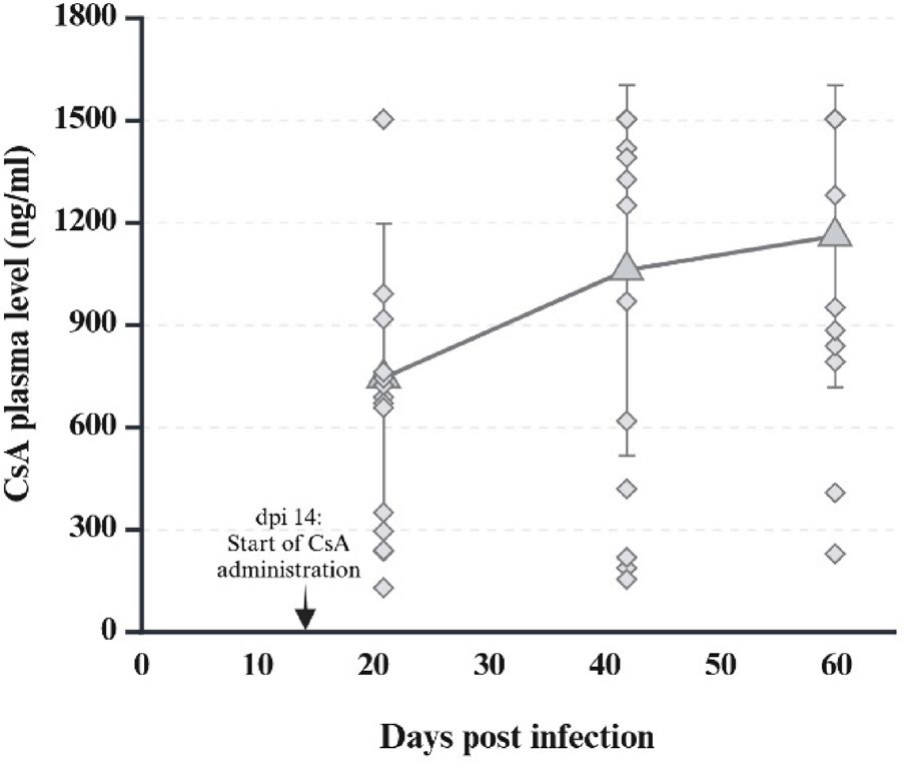
Ciclosporin plasma levels. CsA plasma levels (ng/ml) at 21, 42 and 59/60 dpi, after oral administration of 30 mg/kg Sporimune from 14 dpi onwards. (Created with BioRender.com)

**Fig. 5:**
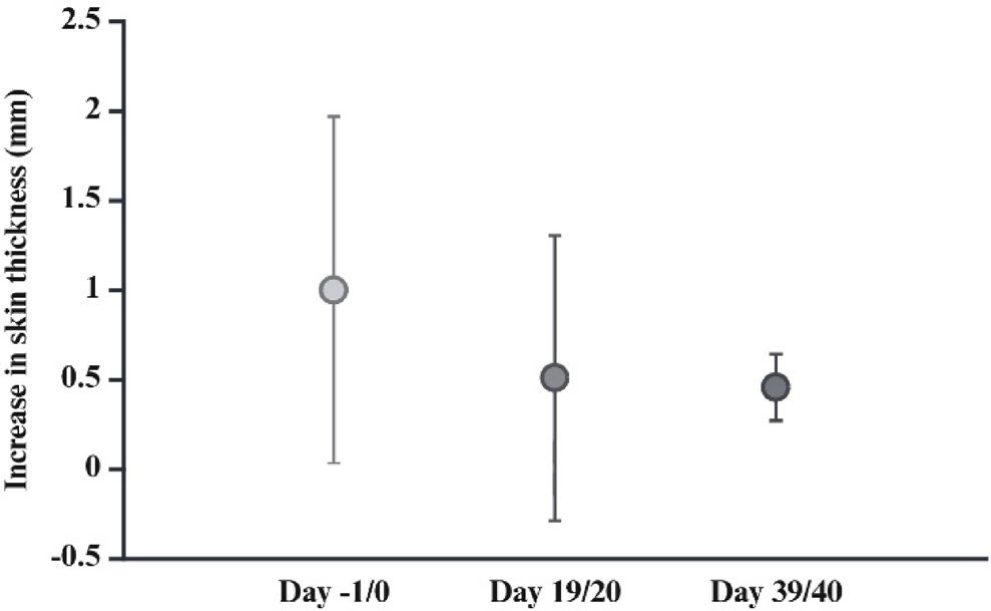
Phytohemagglutinin test. Increase in skin thickness measured on the wingspan 24 h after administration of PHA as an indicator of T-cell mediated immune response at -1/0 dpi (without immunosuppression), 19/20 dpi and 39/40 dpi (under immunosuppression). PHA solved in PBS was injected into the right wingspan, PBS was injected into the left wingspan. The difference between the increase in skin thickness on the right and left sides is estimated as the T-cell mediated immune response. (Created with BioRender.com)

Mean CsA plasma levels were 692 ng/ml (± 469 ng/ml s.d.) at 21 dpi, 1057 ng/ml (± 543 ng/ml s.d.) at 42 dpi and 1157 ng/ml (± 443 ng/ml s.d.) at 59/60 dpi in groups IS and C1, with a minimum value of 150 ng/ml (21 dpi) and maximum values of > 1500 ng/ml (Fig. 4). Low levels of CsA were detected in pigeons not treated with CsA. The mean value of this baseline level in the immunocompetent groups IC and C3 was 38 ng/ml (± 26 ng/ml s.d.) at 59/60 dpi, varying between 14 ng/ml and 105 ng/ml. Based on these data, we propose a baseline cut-off of 120 ng/ml (mean value + standard deviation x 3) for the assessment of CsA plasma levels by mass spectrometry. The phytohemagglutinin assay indicated a marked reduction in the T-cell mediated immune response during CsA treatment. The mean values of the difference PBS/PHA was 1.00 mm (± 0.97 mm s.d.) at 19/20 dpi, 0.51 mm (± 0.8 mm s.d.) at 19/20 dpi and 0.46 mm (± 0.19 mm s.d.) at 39/40 dpi (Fig. 5).

### Clinical signs

In main trial No. 1, pigeons from both groups IS (infected and immunosuppressed) and IC (infected and immunocompetent) showed no clinical signs or altered behaviour during the acute phase. Of the 13 pigeons in each group, only 5 in group IS and 6 in group IC were successfully infected as later determined by histopathology (see below). From 46 dpi onwards, 3 out of 5 successfully infected pigeons in group IS and 4 out of 6 pigeons in group IC developed neurological signs, ranging from mild to severe. 5 pigeons developed mild to moderate ataxia and/or paresis (IS.1, IS.2, IC.6, IC.10, IC.11). One pigeon showed mild to moderate ataxia, torticollis and equilibrium disorders (IC.2) and one pigeon exhibited moderate ataxia and paresis and moderate to severe tumbling, torticollis and equilibrium disorders (IS.10), both were euthanized before the end of the experiment (IS.10 at 53 dpi, IC.2 at 56 dpi). One pigeon displayed mild general signs such as fluffing and closed eyes on 4 individual days (IS.7) and 3 infected pigeons showed no clinical signs during the chronic phase (IS.3, IC.5 & IC.13). Some pigeons in the groups receiving CsA (IS and C1) showed occasional regurgitation (IS.1, IS.2, IS.3, IS.5, IS.10, IS.12, C1.2). Apart from that, all pigeons in the control groups (C1 and C2) showed no clinical signs throughout the trial. No differences in the character or severity of the clinical signs were found between male and female pigeons within the groups. Details of the blinded scorings of clinical signs are displayed in Table 3a.

**Table 3:** Clinical score for neurological signs in the chronic phase of PPE. Unsuccessfully infected pigeons negative for *S. calchasi* by PCR and histopathology did not show neurological signs and are not included in this table. Scores were assigned in a blinded fashion. Pigeons euthanized before the end of the trial were scored as “severe” after euthanasia.

Group	Pigeon No.	Days post infection			
		52	54	56	58
(a) Main trial No. 1.					
IS	IS.1	**2**	**2**	**2**	**2**
	IS.2	**1**	**1**	**1**	**1**
	IS.3	0	0	0	0
	IS.7	0	0	0	0
	IS.10	**2**	**3**	**3**	**3**
IC	IC.2	**1**	**2**	**2**	**3**
	IC.5	0	0	0	0
	IC.6	**1**	**1**	**1**	0
	IC.10	**1**	**1**	**1**	**1**
	IC.11	0	0	0	0
	IC.13	0	0	0	0
C1	C1.1	0	0	0	0
	C1.2	0	0	0	0
	C1.3	0	0	0	0
C2	C2.1	0	0	0	0
	C2.2	0	0	0	0
	C2.3	0	0	0	0
(b) Main trial No. 2.					
			54	56	
AP	AP.1		**1**	**1**	
	AP.2		0	**1**	
	AP.3		**1**	**1**	
	AP.7		**1**	**1**	
	AP.8		0	0	
	AP.10		0	0	
CP	CP.6		0	**1**	
	CP.11		0	0	
	CP.17		0	0	
	CP.18		**1**	**1**	
	CP.19		0	0	
C3	C3.1		0	0	
	C3.2		0	0	
	C3.3		0	0	

IS, immunosuppressed group; IC, immunocompetent group; C1 + 2, control groups; 0, no neurological signs; 1, mild neurological signs; 2, moderate neurological signs; 3, severe neurological signs.

AP, group immunosuppressed on 12-20 dpi; CP, group immunosuppressed from 30 dpi onwards; C3, control group, 0, no neurological signs; 1, mild neurological signs; 2, moderate neurological signs; 3, se-vere neurological signs

In main trial No. 2, all pigeons in groups AP (infected, immunocompromised 12–20 dpi) and CP (infected, immunocompromised from 30 dpi onwards) showed moderate clinical signs and altered behaviour such as fluffing, reduced activity and social interactions polyuria and polydipsia at 10 and 11 dpi before immunosuppression. Twenty-two pigeons from both groups also showed respiratory distress and were euthanized when clinical signs were unlikely to improve. Clinical signs in the remaining pigeons began to improve between 12 and 14 dpi, with some pigeons continuing to show occasional mild fluffing and reduced activity up to 19 dpi. Polyuria and polydipsia improved from 20 dpi onwards and were rarely seen for the remainder of the trial. One pigeon was found dead at 14 dpi (CP.2). Neurological signs started at 45 dpi with six pigeons developing mild equilibrium disorders, ataxia, paresis and/or torticollis (AP.2, AP.3, AP.8, AP.10, CP.11, CP.18). Two pigeons showed mild to moderate ataxia and paresis (AP.1) as well as mild equilibrium disorders (AP.7). Both pigeons had already shown recurrent unsteady walking from 15 dpi (AP.1) and 25 dpi (AP.7) respectively. All pigeons in groups AP and CP showed mild to moderate wing tremors. Again, occasional regurgitation was observed in individual pigeons receiving CsA (AP.1, AP.2 and AP.10 in early phase, CP.6 and CP.18 in late phase). Pigeons in the control group (C3) showed no clinical signs throughout the trial. The blinded clinical scores are summarized in Table 3b.

### Post-mortem macroscopic findings

None of the pigeons examined in the chronic phase of PPE had any macroscopic alterations at necropsy, in agreement with previously published results^18^. Pigeons IS.10 and IC.2 from main trial No. 1 and pigeon AP.7 from main trial No. 2 had streaky patterns in their pectoral muscles. All pigeons euthanized at 10 dpi as well as the pigeon found dead at 14 dpi in main trial No. 2 had moderate to severe splenomegaly and hepatomegaly with multifocal to diffuse pale hepatic necrosis as typical of pigeons dying of *S. calchasi* infection in the acute phase. Bacteriological and parasitological examinations failed to detect any pathogens in the pigeons other than *S. calchasi*.

### Microscopic tissue alterations

#### Main trial 1

Fifteen pigeons in main trial No. 1 that had been inoculated with sporocysts of *S. calchasi* did not contain any sarcocysts in any of the organs examined and no inflammatory lesions in the brain. One pigeon had a lymphohistiocytic inflammation of the skeletal muscles, but no sarcocysts or inflammatory changes in the brain. Sarcocysts were present in the pectoral and femoral muscle of 11 (5 from IS and 6 from IC group) out of 26 infected pigeons in main trial No. 1 (Fig. 2). All successfully infected pigeons euthanized during the chronic phase of PPE (*n* = 11) contained mature sarcocysts in the pectoral and femoral muscles, accompanied by necrosis and inflammation with lymphohistiocytic and eosinophilic cell infiltration. The myocardium of eight pigeons was mildly infested with sarcocysts, in some cases with mild lymphohistiocytic myocarditis. In addition, mild to severe lymphocytic encephalitis with lympho-histiocytic perivascular cuffs, gliosis and varying degrees of necrosis were seen in these pigeons (Fig. 3). However, parasitic structures were not detected in any of the brains histologically. No parasitic structures were detected in any of the other organs examined. No parasitic stages or inflammatory lesions were observed in the control pigeons (groups C1 and C2).

#### Main trial 2

The histopathological findings in the pectoral and femoral muscles and the brain of pigeons euthanized in the chronic phase of PPE (*n* = 11) (6 from AP and 5 from CP group) resembled the pigeons of groups IS and IC in the first main trial (scoring results see below). Again, no parasitic structures were found in the brains. The myocardium of one pigeon was mildly infected with sarcocysts without any inflammation and one pigeon had lymphohistiocytic and eosinophilic myocarditis without any parasitic structures. No parasitic structures were detected in any of the other organs examined. No parasitic stages or inflammatory changes were observed in the control pigeons of group C3.

#### Histological scores

**Fig. 6:**
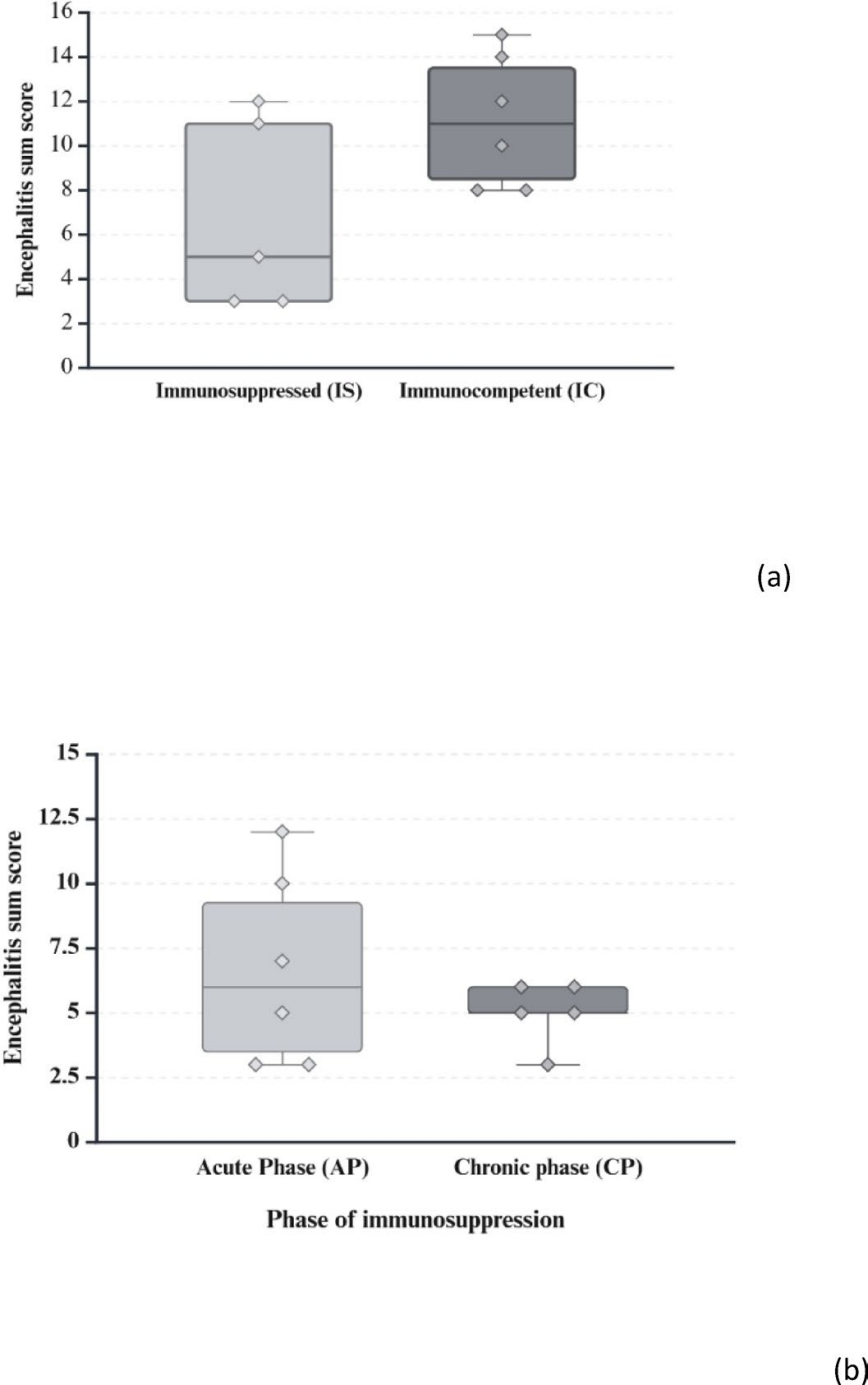
Encephalitis sum scores. Comparison of encephalitis scores. Sum scores are composed of scores for lymphohistiocytic encephalitis, perivascular immune cell cuffs and gliosis. (Created with BioRender.com). (**a**) Main trial No. 1: Comparison between the immunosuppressed (IS) and immunocompetent (IC) group infected with *S. calchasi*. (**b**) Main trial No. 2: Comparison between immunosuppression pigeons during the early (AP) or late (CP) phase of *S. calchasi* infection.

Semi quantification of the severity of microscopic brain lesions by scoring was performed on the successfully infected animals, incidentally uninfected animals were excluded. Encephalitis scores (sum of scores for lymphohistiocytic encephalitis, perivascular immune cell cuffs and gliosis) in main trial No. 1 varied between 3 and 12 in group IS (infected, immunocompromised) with a mean score of 6.8 (± 4.4 s.d.), and between 8 and 15 in group IC (infected, immunocompetent) with a mean of 11.2 (± 3.0 s.d.) (Table 4; Fig. 6a). The differences between the groups almost reached statistical significance (*p* = 0.069).

**Table 4:** Histopathological scores in the brain of pigeons euthanized in chronic phase of PPE. Unsuccessfully infected pigeons that were negative for *S. calchasi* by PCR and histopathology did not show neurological signs and are not included in this table. Scores were assigned in a blinded fashion.

Group	Pigeon No.	Immune cell infiltration/Inflammation				
		Lympho-histiocytic perivascular cuffs	Lymphocytic encephalitis	Gliosis	Sum score	Necrosis
(a) Main trial No. 1						
IS	IS.1	1	2	2	**5**	2
	IS.2	4	4	4	**12**	2
	IS.3	1	1	1	**3**	0
	IS.7	1	1	1	**3**	1
	IS.10	5	4	2	**11**	4
IC	IC.2	3	3	2	**8**	5
	IC.5	2	3	3	**8**	3
	IC.6	5	4	5	**14**	5
	IC.10	5	5	5	**15**	3
	IC.11	4	4	4	**12**	3
	IC.13	2	4	4	**10**	2
C1	C1.1	0	0	0	0	0
	C1.2	0	0	0	0	0
	C1.3	0	0	0	0	0
C2	C2.1	0	0	0	0	0
	C2.2	0	0	0	0	0
	C2.3	0	0	0	0	0
(b) Main trial No. 2						
AP	AP.1	4	4	4	**12**	1
	AP.2	1	1	1	**3**	1
	AP.3	1	1	1	**3**	1
	AP.7	2	2	3	**7**	1
	AP.8	4	3	3	**10**	3
	AP.10	1	2	2	**5**	1
CP	CP.6	1	2	2	**5**	1
	CP.11	2	2	2	**6**	2
	CP.17	2	2	2	**6**	1
	CP.18	1	2	2	**5**	1
	CP.19	1	1	1	**3**	1
C3	C3.1	0	0	0	0	0
	C3.2	0	0	0	0	0
	C3.3	0	0	0	0	0

IS, immunosuppressed group; IC, immunocompetent group; C1 + 2, control groups; 1, 0–20%; 2, 20–40%; 3, 40–60%; 4, 60–80%; 5, 80–100%. AP, group immunosuppressed on 12–20 dpi; CP, group immunosuppressed from 30 dpi onwards; C3, control group; 1, 0–20%; 2, 20–40%; 3, 40–60%; 4, 60–80%; 5, 80–100%.

**Fig. 7:**
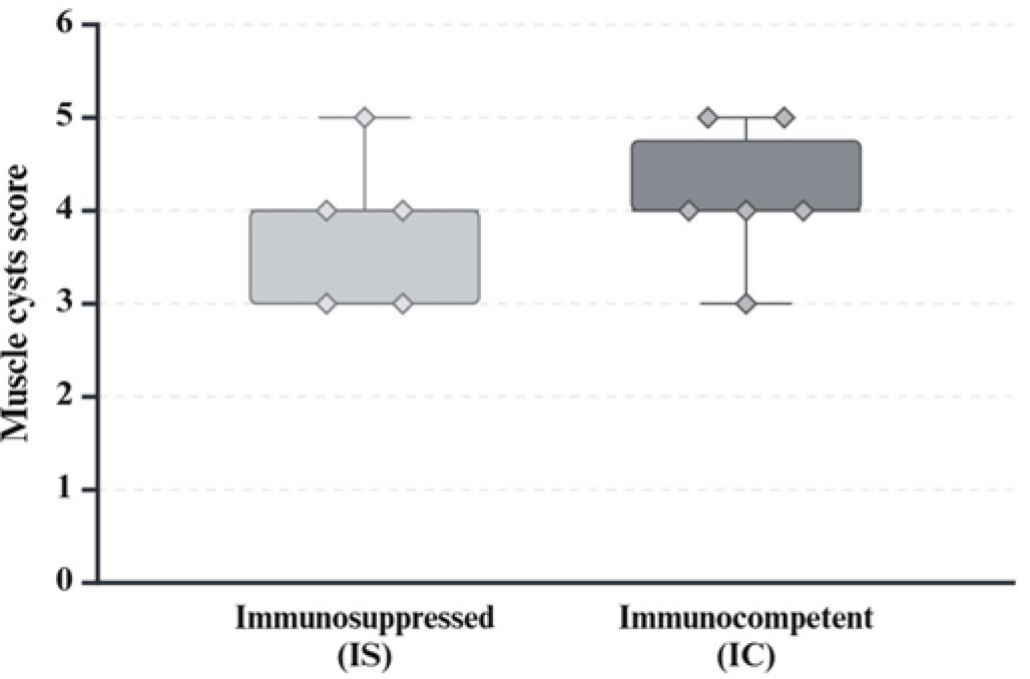
Sarcocyst score in striated musculature. Comparison of sarcocyst score between the immunosuppressed (IS) and immunocompetent (IC) groups infected with *S. calchas*i. (Created with BioRender.com)

The quantity score of cysts in skeletal muscles varied between 3 and 5 in both groups IS and IC, with means of 3.8 (± 0.84 s.d.) in group IS and 4.2 (± 0.75 s.d.) in group IC, respectively (*p* = 0.35) (Fig. 7).

In main trial No. 2 encephalitis sum scores varied between 3 and 12 in group AP (infected, immunocompromised 12–20 dpi) with a mean of 6.7 (± 3.7 s.d.) and between 3 and 6 in group CP (infected, immunocompromised from 30 dpi onwards) with a mean of 5 (± 1.2 s.d.). Results from statistical testing were far from indicating differences between groups AP and CP (*p* = 0.63) (Table 4; Fig. 6b).

### Immunohistochemistry

**Fig. 8:**
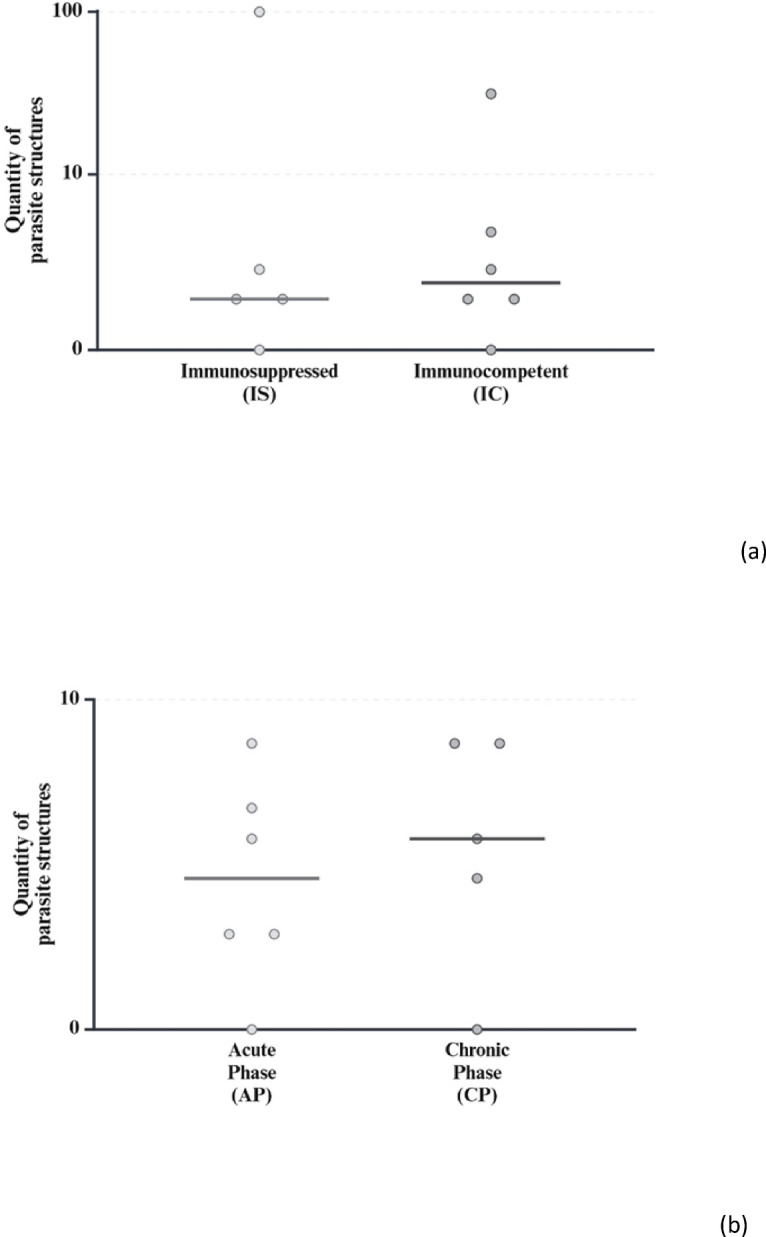
Quantity of parasite structures detected by immunohistological analysis in brains of pigeons experimentally infected with *S. calchasi*. (Created with BioRender.com). (**a**) Main trial No. 1, (**b**) Main trial No. 2.

**Fig. 9:**
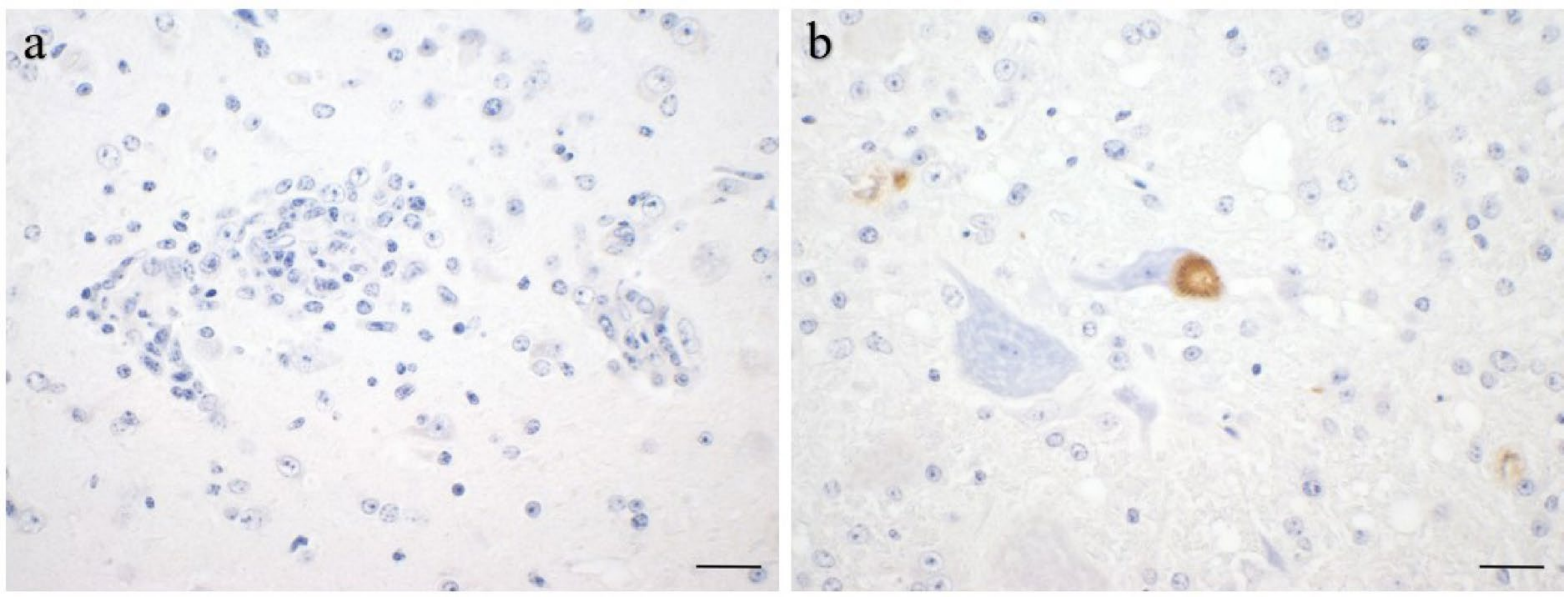
Immunohistochemical detection of *S. calchasi* antigen in brains of experimentally infected pigeons. (**A**): Lymphohistiocytic infiltration and glial cell proliferation with no parasitic antigen (IS.10). (**B**): Intraneuronal parasitic structures consistent with schizonts with no evidence of associated inflammation (IS.10) Brown: *S. calchasi* antigen visualized by diaminobenzidine (DAB) with Mayer’s hemalaun counterstain (blue), scale bars: 20 μm.

Despite impressive inflammatory changes, most brains from all groups in main trial No. 1 contained no or only very little parasitic antigen (Fig. 8a). Even in those pigeons with a few parasitic structures, these were spatially not associated with encephalitis, with the vast majority of inflamed areas lacking any evidence of parasitic antigen (Fig. 9a). However, in both groups IS and IC one animal each had a high number of immunohistochemically labelled parasitic structures consistent with *S. calchasi* schizonts and merozoites (IS.10, IC.11). Of note, even in these two animals most pathogens were not directly associated with inflammatory lesions (Fig. 9b). Overall, we failed to detect any difference between groups IS and IC in terms of numbers and distribution of parasitic antigens, with no evidence of any effect of immunosuppression.

Similar results were obtained in main trial No. 2 with only very few if any parasitic structures in the brains, again most of them not spatially associated with inflammatory lesions **(**Fig. 8b**)**. In contrast to groups IS and IC, none of the pigeons had a high amount of parasitic antigen in their brains.

### Detection of* Sarcocystis calchasi *DNA

All pigeons of both main trials with histologically visible sarcocysts characteristic of *Sarcocystis* spp. were positive by *S. calchasi*-specific PCR in the brain and skeletal muscle samples (Table 5). All pigeons from main trial No. 1 that did not contain sarcocysts as detected by histology tested negative by PCR. All pigeons from main trial No. 2 that were euthanized at 10 dpi due to severe clinical signs tested positive for *S. calchasi* by PCR in their livers. *Sarcocystis calchasi* DNA was not detected in any of the samples from the control pigeons.

**Table 5:** Results of *S. calchasi*-specific PCR.

Group	Pigeon No.	Dpi	Organs		
			Brain	Skeletal muscle	Liver
(a) Main trial No. 1					
IS	IS.1	59	**pos**	**pos**	nd
	IS.2	59	**pos**	**pos**	nd
	IS.3	59	**pos**	**pos**	nd
	IS.4	59	neg	neg	nd
	IS.5	59	neg	neg	nd
	IS.6	59	neg	neg	nd
	IS.7	60	**pos**	**pos**	nd
	IS.8	60	neg	neg	nd
	IS.9	60	neg	neg	nd
	IS.10	53	**pos**	**pos**	nd
	IS.11	60	neg	neg	nd
	IS.12	60	neg	neg	nd
	IS.13	60	neg	neg	nd
IC	IC.1	59	neg	neg	nd
	IC.2	56	**pos**	**pos**	nd
	IC.3	59	neg	neg	nd
	IC.4	59	neg	neg	nd
	IC.5	59	**pos**	**pos**	nd
	IC.6	59	**pos**	**pos**	nd
	IC.7	59	neg	neg	nd
	IC.8	60	neg	neg	nd
	IC.9	60	neg	neg	nd
	IC.10	60	**pos**	**pos**	nd
	IC.11	60	**pos**	**pos**	nd
	IC.12	60	neg	neg	nd
	IC.13	60	**pos**	**pos**	nd
C1	C1.1	59	neg	neg	nd
	C1.2	59	neg	neg	nd
	C1.3	59	neg	neg	nd
C2	C2.1	59	neg	neg	nd
	C2.2	59	neg	neg	nd
	C2.3	59	neg	neg	nd
(b) Main trial No. 2					
AP	AP.1	60	**pos**	**pos**	nd
	AP.2	60	**pos**	**pos**	nd
	AP.3	60	**pos**	**pos**	nd
	AP.4	10	nd	nd	**pos**
	AP.5	10	nd	nd	**pos**
	AP.6	10	nd	nd	**pos**
	AP.7	60	**pos**	**pos**	nd
	AP.8	60	**pos**	**pos**	nd
	AP.9	10	nd	nd	**pos**
	AP.10	60	**pos**	**pos**	nd
	AP.11	10	nd	nd	**pos**
	AP.12	10	nd	nd	**pos**
	AP.13	10	nd	nd	**pos**
	AP.14	10	nd	nd	**pos**
	AP.15	10	nd	nd	**pos**
	AP.16	10	nd	nd	**pos**
CP	CP.1	10	nd	nd	**pos**
	CP.2	14	**pos**	nd	**pos**
	CP.3	10	nd	nd	**pos**
	CP.4	10	nd	nd	**pos**
	CP.5	10	nd	nd	**pos**
	CP.6	60	**pos**	**pos**	nd
	CP.7	10	nd	nd	**pos**
	CP.8	10	nd	nd	**pos**
	CP.9	10	nd	nd	**pos**
	CP.10	10	nd	nd	**pos**
	CP.11	60	**pos**	**pos**	nd
	CP.12	10	nd	nd	**pos**
	CP.15	10	**pos**	nd	**pos**
	CP.17	60	**pos**	**pos**	nd
	CP.18	60	**pos**	**pos**	nd
	CP.19	60	**pos**	**pos**	nd
C3	C3.1	60	neg	neg	nd
	C3.2	60	neg	neg	nd
	C3.3	60	neg	neg	nd

IS, immunosuppressed group; IC, immunocompetent group; C1 + 2, control groups; Dpi, Days post infection; neg, negative; pos, positive, nd, not done. AP, group immunosuppressed on 12–20 dpi; CP, group immunosuppressed from 30 onwards dpi; C3, control group.

## Discussion

*Sarcocystis calchasi* has recently been identified as the cause of a severe neurological disease in pigeons. It causes a biphasic disease with an acute phase characterized by transient nonspecific general disease followed by a chronic phase of severe, progressive neurological disorders associated with granulomatous encephalitis^18^. As lesion-associated parasitic structures are rarely found in these brains, the underlying pathomechanism of the encephalitis is currently unknown. Previous studies have suggested that the encephalitis is subject to an immunomodulation caused by *S. calchasi* as a delayed hypersensitivity reaction. However, an alternative scenario with only early parasitic invasion of the brain as a hit-and-run mechanism has also been discussed^17,20^. Therefore, the aim of the present study was to test whether an immunopathogenesis may be triggered by early or late parasitic stages. We thus hypothesized that experimental immunosuppression would alleviate the course of the disease, possibly in a stage-dependent manner.

The experiments were designed to induce the typical biphasic disease in the pigeons infected with *S. calchasi*, as originally described by *Olias* et al.^18^. Unexpectedly, the infectious dose of 200 sporocysts per pigeon resulted in successful infection of only 42% of the pigeons in main trial No. 1, despite the successful infection of all pigeons of the pre-trial with the same dose. Internal reviews of the methods used for infection did not reveal any errors that could have led to the failure of infection in the majority of pigeons. Since a decrease in viability and infectivity has been previously observed in *S. calchasi* sporocysts from the Berlin strain after a longer period of storage^17^, it could not be excluded that the sporocysts had begun to lose viability between the preliminary and main trial. Therefore, a host passage was performed after main trial No. 1. This resulted in a 100% infection rate in main trial 2, supporting the hypothesis of an initial loss of viability before. However, the post-passage infection dose of 100 sporocysts per pigeon in main trial No. 2, which was only half of the previous dose in main trial 1, resulted in severe clinical signs in 63% of the infected pigeons during the acute phase, although a dose of 200 sporocysts had been suggested to be safe in the pre-trial, which had ended one week earlier. Both deviations suggest that changes in infectivity and virulence may occur during storage of sporocysts at 4 °C, as observed in previous studies^17^, even with natural host passage. This again highlights the importance of pre-trials to determine the appropriate infectious dose in studies with *S. calchasi*, still with the potential for further increase or loss of virulence by the time the main trial begins. Interestingly, a decrease in infectivity and virulence has been demonstrated in *S. neurona* sporocysts under equal conditions during long-term storage and similar results of reduced infectivity have been reported for *S. cruzi* sporocysts in cell culture^26,27^. In contrast, studies using nematode parasites such as *Steinernema scarabaei* have observed an increase in virulence and infectivity over time at a storage temperature of 8 °C, while a decrease was detected at room temperature^28^.The reasons for the observed changes in virulence of *S. calchasi* are currently unknown. Whether the parasite uses mechanisms to control its virulence or what other factors may be involved should be addressed in further studies.

CsA has already been used in several studies to selectively suppress T-cell function in chickens^29–31^, turkeys^32–34^, pheasants^35^ and cockatiels^36^. To the authors´ knowledge this effect has not been investigated in domestic pigeons to date and the use of Sporimune in birds has not been published before. CsA plasma concentrations were therefore monitored to control for effective absorption. Oral administration in this study resulted in noticeably higher plasma levels than previously published for either the oral (33 ng/ml) or intramuscular routes (540 ± 246 ng/ml s.d.), although blood samples were taken between 20 and 24 h after the last oral dose and plasma levels have been described to peak around 1 h after administration^30^. Mean levels increased throughout the experiment suggesting some degree of slow absorption with transient accumulation of CsA. No data is currently available on therapeutically effective plasma levels of CsA in birds. Comparison with other species shows that 250 to 500 ng/ml in cats and 500 ng/ml in dogs are described as target plasma levels when used as an immunosuppressant^37^. In the current study, these levels were exceeded by 38% of CsA-treated pigeons at dpi 21, by 111% at dpi 42 and by 131% at dpi 59/60, suggesting that CsA levels were effective for the purpose of this study.

The PHA test was used to assess the effect of CsA on T-cell function in the pigeons. PHA tests have been used in vivo in several avian species to measure T-cell mediated immunity and immunodeficiency^24,38–41^. Our PHA data indicated a lower increase in skin thickness at the two time points under immunosuppression when compared to immunocompetent pigeons, further supporting the effective suppression of the T-cell activity in the CsA-treated pigeons.

Some pigeons showed occasional regurgitation during the treatment with CsA. Mild gastrointestinal signs such as regurgitation or vomiting have been reported to be the most common adverse reactions following oral administration of CsA in dogs and cats^42–45^. Further investigation is needed to test whether lower doses of oral CsA can achieve similar plasma levels and T-cell suppression in birds with fewer side effects.

The results of pre-trial No. 1.2 demonstrated that immunosuppression during the acute phase of a S. calchasi infection leads to increased lethality. Contrary to the chronic phase, the acute phase of PPE is most likely mediated by direct host-pathogen interaction with multiple schizogonies leading to massive destruction of liver parenchyma^18^. Additionally, host immune response is downregulated during this phase, most probably due to immune evasion strategies of the parasite^20^. Further suppression of T-cell function by ciclosporin application most likely enhances parasite invasion and destruction of host tissue thus leading to an increased lethality during the acute phase.

Clinical signs during the late phase of PPE did not differ in character or severity between immunocompromised and immunocompetent animals. Neurological signs such as ataxia, paresis, tumbling, torticollis and equilibrium disorders were observed to varying degrees in all infected groups. There was no correlation between the severity of clinical signs and the degree of encephalitis in the individual pigeons as has been shown previously^17^. It is most likely that the severity and character of neurological signs in individual birds depends largely on the distribution and location of brain lesions, which have not been further investigated in this study. Encephalitis in the course of PPE has been reported to randomly affect all areas of the brain^19^. The spectrum of neurological disorders observed in our study strongly suggests involvement of the cerebrum, cerebellum, brainstem and the vestibular system, as previously observed^46^.

In general, our histopathological findings resemble those of previous studies, including consistent and marked global encephalitis despite no or minimal numbers of parasitic stages in the brains^17^. However, immunohistochemistry revealed high numbers of schizonts and merozoites in the brain of one animal in each of the groups IS and IC, both euthanized in the chronic phase of PPE, and individual parasite stages in the other pigeons in both main trials. This contrasts with the results of previous infection trials, where rare schizonts were found in the brain tissue of half of the pigeons, but only of those that died in the acute phase of PPE, while sarcocysts were found in unaffected brain areas in some of the pigeons in the chronic phase of PPE^17,20^. One study has reported *S. calchasi* schizonts, even associated with brain lesions, in the brains of naturally infected pigeons in North America^9^. However, it cannot be excluded that the schizonts in this case were due to reinfection or strain differences, which can be ruled out in our study. The reason for the presence of schizonts in the brains of the two pigeons remains unclear, but an association with immunosuppression seems unlikely, as pigeons in both groups were affected. Regarding the lack of a direct association between the majority of parasite stages and inflammatory brain lesions, our results are similar to those of previous studies^17,20^ Of note, in main trial No. 1, histological encephalitis scores were clearly higher in the immunocompetent group (IC) compared to the immunocompromised group (IS), although the p-value was slightly above the significance level (0.069). Nevertheless, immunosuppression during a *S. calchasi* infection seems to ameliorate the severity of encephalitis, suggesting that the encephalitis during PPE is subject to a stimulation of the immune response, especially involving T-cells. It cannot be excluded that the parasitic stages prior to the initiation of CsA treatment at 14 dpi did already induce an immune response, and that the effect of immunosuppression in ameliorating the severity of encephalitis would have been greater if immunosuppression had been initiated earlier after infection. However, as such early immunosuppression resulted in a fatal outcome of the infection during the acute phase, we refrained from further pursuing this notion. In addition, we speculate that the results would have been more statistically distinct with the originally planned number of pigeons per group, which was inadvertently reduced by unexpected and unexplained losses. A possible cause could be genetic heterogeneity or different immunological status due to unknown historical events in some of the animals. This could also explain the higher numbers of immunohistological detected parasitic stages in two pigeons (Fig. 8). Unfortunately, genetically homogeneous pigeons reared under highly standardized conditions are scarce. Spare animals should be included in further studies when possible, to obtain the appropriate number of infected animals.

Studies on the pathogenesis of Parrot Bornavirus, the causative agent of Proventricular Dilatation Disease (PDD), in cockatiels have used a similar experimental design to test the possible role of a T-cell mediated disease^36^. Similar results to our study were obtained, with birds infected with Parrot Bornavirus and treated with CsA developing fewer microscopic lesions when compared to non-CsA treated birds, although similarly high levels of viral RNA were detected in all birds^36^. Furthermore, a study in horses infected with the closely related *S. neurona*, the causative agent of Equine Protozoal Myeloencephalitis (EPM), and suffering from SCID (severe combined immune deficiency), a disease which is characterized by a lack of specific B- and T-cell responses, showed that these horses did not develop the typical neurological signs, neurological lesions or neuroinvasion of the parasite, despite prolonged parasitemia and persistent infection in visceral tissues, whereas immunocompetent control horses developed neurological signs^47^. Both studies emphasize the possibility of an exaggerated immune response as a cause of neurological lesions, as suggested by our study for *S. calchasi*. Autoimmune pathomechanisms triggering encephalitis are also known in numerous human diseases. For instance, Acute Disseminated Encephalomyelitis (ADEM) is an immune-mediated inflammatory disorder of the central nervous system that is usually preceded by a viral infection or vaccination^48^, but has also been observed following parasitic infection with *Plasmodium vivax*^49^, and is characterized by the absence of pathogenic antigen within the central nervous system. Again, T- cells are suspected to be the primary mediators of disease^50^.

In contrast to main trial No. 1, histological scores in main trial 2 showed no difference between the two trial groups. Therefore, our results suggest that immunosuppression only during the early phase of infection, immediately after the acute phase but before the appearance of immature sarcocysts or any encephalitic lesions, had the same effect as immunosuppression from 30 dpi onwards. This suggests that the immune-mediated encephalitis is likely to be triggered particularly at an early stage of infection, probably by sporozoites and/or merozoites. If immature cysts had played a role in triggering an auto-directed immune response, the effects of immunosuppression during the early phase would have been much less pronounced than during the late phase. We therefore speculate that merozoites, as the predominant developmental stage of *S. calchasi* in the acute phase of PPE, may act as the major trigger of encephalitis in PPE. This would explain why a complete prevention of encephalitis could not be achieved in both main trials, because CsA treatment could not be started until after the clinical signs of the acute phase had diminished. As mentioned above, it would be necessary to study immunocompromised pigeons before and during the acute phase, when merozoites are likely to be the predominant parasitic stage, which does not seem possible at present.

In infections with *Sarcocystis* spp., sporozoites and merozoites have been shown to be the immunodominant stage during the development of the parasite in its intermediate hosts, for example in studies on immunization with sporocysts of different *Sarcocystis* species in goats, pigs and cattle^51–54^: Animals infected with low doses of infectious sporocysts and then challenged with a lethal or pathogenic dose of sporocysts of the homologous *Sarcocystis* species developed reduced clinical signs compared to the controls. In contrast, immunization of mice and pigs with cystozoites or cystozoite fractions of *Sarcocystis* spp. resulted in the production of specific antibodies, but without the formation of protective immunity^55,56^. Therefore, it can be assumed that protective immunity against *Sarcocystis* spp. is induced by sporozoites and/or merozoites, and the same may be true for a misdirected immune response provoking encephalitis as shown here in pigeons infected with *S. calchasi*.

In conclusion, the results of this study support the hypothesis of immunomodulation by *S. calchasi* in the development of encephalitis in the course of PPE. It is suggested that sporozoites and/or merozoites act as parasitic stages interacting with the immune system of pigeons as intermediate hosts. Overall, our data suggest that the encephalitis in the late phase of *S. calchasi* infection is more likely to be triggered by a T-cell mediated immune mechanism leading to a delayed-type hypersensitivity response rather than by a destructive hit-and-run mechanism directly induced by parasitic stages.

## Data Availability

The datasets generated during the current study are available from the corresponding author on reasonable request.

## References

[1] Olias, P. et al. *Sarcocystis calchasi* sp. Nov. Of the domestic pigeon (*Columba Livia* f. *domestica*) and the Northern goshawk (*Accipiter gentilis*): light and electron microscopical characteristics. *Parasitol. Res.* **106**, 577–585. 10.1007/s00436-009-1701-9 (2009). 20033211 10.1007/s00436-009-1701-9

[2] Olias, P., Olias, L., Krücken, J., Lierz, M. & Gruber, A. D. High prevalence of sarcocystis calchasi sporocysts in European accipiter Hawks. *Vet. Parasitol.* **175**, 230–236 (2011). 21074324 10.1016/j.vetpar.2010.10.025

[3] Rogers, K. H., Arranz-Solís, D., Saeij, J. P., Lewis, S. & Mete, A. Sarcocystis calchasi and other sarcocystidae detected in predatory birds in california, USA. *Int. J. Parasitol. Parasites Wildl.* **17**, 91–99 (2022). 35004169 10.1016/j.ijppaw.2021.12.008 PMC8718662

[4] Olias, P. et al. Sarcocystis species lethal for domestic pigeons. *Emerg. Infect. Dis.* **16**, 497 (2010). 20202429 10.3201/eid1603.090860 PMC3322016

[5] Hodo, C. L. et al. Histopathologic and molecular characterization of sarcocystis calchasi encephalitis in white-winged doves (Zenaida asiatica) and Eurasian collared doves (Streptopelia decaocto), East-central texas, USA, 2010–13. *J. Wildl. Dis.* **52**, 395–399 (2016). 27124332 10.7589/2015-10-292 PMC4889117

[6] Parmentier, S. L. et al. Prevalence of sarcocystis calchasi in free-ranging host species: accipiter Hawks and common woodpigeon in Germany. *Sci. Rep.* **8**, 1–8 (2018). 30514865 10.1038/s41598-018-35862-x PMC6279811

[7] Parmentier, S. L., Maier-Sam, K., Failing, K., Gruber, A. D. & Lierz, M. High prevalence of sarcocystis calchasi in racing pigeon flocks in Germany. *PLoS One*. **14**, e0215241 (2019). 30986233 10.1371/journal.pone.0215241 PMC6464325

[8] Rimoldi, G. et al. An outbreak of *Sarcocystis calchasi* encephalitis in multiple Psittacine species within an enclosed zoological aviary. *J. Vet. Diagn. Invest.* **25**, 775–781. 10.1177/1040638713502981 (2013). 24081928 10.1177/1040638713502981

[9] Olias, P. et al. Sarcocystis calchasi has an expanded host range and induces neurological disease in cockatiels (Nymphicus hollandicus) and North American rock pigeons (Columbia Livia f. dom). *Vet. Parasitol.* **200**, 59–65 (2014). 24360290 10.1016/j.vetpar.2013.11.012

[10] Bamac, O. E. et al. Protozoal encephalitis associated with Sarcocystis calchasi and S. falcatula during an epizootic involving Brandt’s cormorants (Phalacrocorax penicillatus) in coastal Southern California, USA. *Int J. Parasitol. Parasites Wildl* (2020). 10.1016/j.ijppaw.2020.06.005 PMC7322481 32617260

[11] Ziegler, L. et al. Investigations into causes of neurologic signs and mortality and the first identification of *Sarcocystis calchasi* in free-ranging woodpeckers in Germany. *J. Zoo Wildl. Med.* **49**, 247–251. 10.1638/2017-0087r.1 (2018). 29517425 10.1638/2017-0087R.1

[12] Gadsby, S. et al. Fatal sarcocystis calchasi–associated meningoencephalitis in 2 captive vulturine guineafowl. *J. Vet. Diagn. Invest.* **34**, 543–546 (2022). 35168421 10.1177/10406387221078585 PMC9254049

[13] Prakas, P., Calero-Bernal, R. & Dubey, J. P. Sarcocystis infection in domestic and wild avian hosts: inseparable flight partners. *Vet Parasitol*, 110413 (2025). 10.1016/j.vetpar.2025.110413 40023975

[14] Dubey, J. P., Speer, C. A. & Fayer, R. *Sarcocystosis of Animals and Man* (CRC Press, Inc., 1988).

[15] Mehlhorn, H. & Heydorn, A. O. The sarcosporidia (Protozoa, Sporozoa): life cycle and fine structure. *Adv. Parasitol.* **16**, 43–91 (1978). 103377 10.1016/s0065-308x(08)60572-2

[16] Olias, P., Olias, L., Lierz, M., Mehlhorn, H. & Gruber, A. D. Sarcocystis calchasi is distinct to sarcocystis columbae sp. Nov. From the wood pigeon (Columba palumbus) and sarcocystis sp. From the Sparrowhawk (Accipiter nisus). *Vet. Parasitol.* **171**, 7–14 (2010). 20381254 10.1016/j.vetpar.2010.03.021

[17] Maier, K. et al. Parasite distribution and early stage encephalitis in *Sarcocystis calchasi* infections in domestic pigeons (*Columba Livia* f. *domestica*). *Avian Pathol.* **44**, 5–12. 10.1080/03079457.2014.978263 (2015). 25338141 10.1080/03079457.2014.978263

[18] Olias, P. et al. Unusual biphasic disease in domestic pigeons (*Columba Livia* f. *domestica*) following experimental infection with *Sarcocystis calchasi*. *Avian Dis.* **54**, 1032–1037. 10.1637/9303-031110-Reg.1 (2010). 20945785 10.1637/9303-031110-Reg.1

[19] Olias, P. et al. A novel *Sarcocystis*-associated encephalitis and myositis in racing pigeons. *Avian Pathol.* **38**, 121–128. 10.1080/03079450902737847 (2009). 19322710 10.1080/03079450902737847

[20] Olias, P. et al. Modulation of the host Th1 immune response in pigeon protozoal encephalitis caused by sarcocystis calchasi. *Vet. Res.* **44**, 10 (2013). 23398807 10.1186/1297-9716-44-10 PMC3598538

[21] Rommel, M. et al. Office for official publications on the European Communities,. In *COST 89/820: Biotechnology: Guidelines on techniques in coccidiosis research.* (eds J. Eckert, R. Braun, M. W. Shirley, & P. Coudert) 241–284 (1995).

[22] Milićević, Ž., Živanović, V. & Milićević, N. Involution of bursa cloacalis (Fabricii) and thymus in cyclosporin A-Treated chickens. *Anat. Histol. Embryol.* **31**, 61–64 (2002). 11841358 10.1046/j.1439-0264.2002.00358.x

[23] Smits, J., Bortolotti, G. R. & Tella, J. L. Simplifying the phytohaemagglutinin skin-testing technique in studies of avian immunocompetence. *Funct. Ecol.* **13**, 567–572 (1999).

[24] Fair, J. M., Hansen, E. S. & Ricklefs, R. E. Growth, developmental stability and immune response in juvenile Japanese quails (Coturnix coturnix japonica). *Proc. R. Soc. Lond., Ser. B: Biol. Sci.* 266, 1735–1742,10.1098/rspb.1999.0840 (1999). 10.1098/rspb.1999.0840 PMC1690192 10518322

[25] Dietert, K. et al. Murine CLCA5 is uniquely expressed in distinct niches of airway epithelial cells. *Histochem. Cell. Biol.* **143**, 277–287. 10.1007/s00418-014-1279-x (2015). 25212661 10.1007/s00418-014-1279-x PMC4317516

[26] Elsheikha, H., Murphy, A. & Mansfield, L. Viability of sporocysts after long-term storage. *Vet. Parasitol.* **123**, 257–264. 10.1016/j.vetpar.2004.06.013 (2004). 15325051 10.1016/j.vetpar.2004.06.013

[27] Savini, G., Robertson, I. D. & Dunsmore, J. D. Excystation rates and infectivity of sporocysts of sarcocystis Cruzi exposed to different treatments and storages. *Vet. Parasitol.* **73**, 17–25 (1997). 9477488 10.1016/s0304-4017(97)00068-x

[28] Koppenhöfer, A. M., Ebssa, L. & Fuzy, E. M. Storage temperature and duration affect Steinernema scarabaei dispersal and attraction, virulence, and infectivity to a white Grub host. *J. Invertebr Pathol.* **112**, 129–137 (2013). 23201455 10.1016/j.jip.2012.11.002

[29] Wick, G., Müller, P. U. & Schwarz, S. Effect of cyclosporin A on spontaneous autoimmune thyroiditis of obese strain (OS) chickens. *Eur. J. Immunol.* **12**, 877–881 (1982). 6756938 10.1002/eji.1830121014

[30] Nowak, J. S., Osamu, K., Peck, R. & Franklin, R. M. The effects of cyclosporin A on the chicken immune system. *Eur. J. Immunol.* **12**, 867–876 (1982). 6983442 10.1002/eji.1830121013

[31] Raj, G. D. & Jones, R. Effect of T-cell suppression by cyclosporin on primary and persistent infections of infectious bronchitis virus in chickens. *Avian Pathol.* **26**, 257–276 (1997). 18483906 10.1080/03079459708419210

[32] Rubbenstroth, D., Dalgaard, T. S., Kothlow, S., Juul-Madsen, H. R. & Rautenschlein, S. Effects of cyclosporin A induced T-lymphocyte depletion on the course of avian metapneumovirus (aMPV) infection in Turkeys. *Dev. Comp. Immunol.* **34**, 518–529 (2010). 20043941 10.1016/j.dci.2009.12.011

[33] Khehra, R. & Jones, R. Investigation into avian Pneumovirus persistence in Poults and chicks using cyclosporin A immunosuppression. *Res. Vet. Sci.* **66**, 161–163 (1999). 10208895 10.1053/rvsc.1998.0257

[34] Suresh, M. & Sharma, J. M. Hemorrhagic enteritis virus induced changes in the lymphocyte subpopulations in Turkeys and the effect of experimental immunodeficiency on viral pathogenesis. *Vet. Immunol. Immunopathol.* **45**, 139–150 (1995). 7604531 10.1016/0165-2427(94)05323-k

[35] Fitzgerald, S., Reed, W., Furukawa, A., Zimels, E. & Fung, L. Effect of T-lymphocyte depletion on the pathogenesis of marble spleen disease virus infection in ring-necked pheasants. *Avian Dis.*, 68–73 (1995). 7794193

[36] Hameed, S. S., Guo, J., Tizard, I., Shivaprasad, H. & Payne, S. Studies on immunity and Immunopathogenesis of Parrot Bornaviral disease in cockatiels. *Virology* **515**, 81–91 (2018). 29274528 10.1016/j.virol.2017.12.007

[37] Plumb, D. C. *Plumb’s Veterinary Drug Handbook* 7 edn (Wiley-Blackwell, 2011).

[38] GOTO, N., OKADA, K. O. D. A. M. A. H., FUJIMOTO, Y. & K. & Suppression of Phytohemagglutinin skin response in thymectomized chickens. *Poultl Sci.* **57**, 246–250 (1978). 10.3382/ps.0570246 674011

[39] McCorkle, F., Simmons, D. & Luginbuhl, G. Delayed hypersensitivity response in alcaligenes faecalis-infected Turkey Poults. *Avian Dis.*, 782–786 (1982). 7159321

[40] Tella, J. L. et al. Offspring body condition and immunocompetence are negatively affected by high breeding densities in a colonial seabird: a multiscale approach. *Proc. R Soc. Lond. Ser. B: Biol. Sci.* **268**, 1455–1461. 10.1098/rspb.2001.1688 (2001). 10.1098/rspb.2001.1688 PMC1088763 11454288

[41] Gethöffer, F. et al. The modulating effect of food composition on the immune system in growing ring-necked pheasants (Phasianus colchicus). *PLoS One*. **17**, e0277236 (2022). 36342931 10.1371/journal.pone.0277236 PMC9639844

[42] Allenspach, K. et al. Pharmacokinetics and clinical efficacy of cyclosporine treatment of dogs with steroid-refractory inflammatory bowel disease. *J. Vet. Intern. Med.* **20**, 239–244 (2006). 16594578 10.1892/0891-6640(2006)20[239:paceoc]2.0.co;2

[43] Steffan, J., Alexander, D., Brovedani, F. & Fisch, R. D. Comparison of cyclosporine A with Methylprednisolone for treatment of canine atopic dermatitis: a parallel, blinded, randomized controlled trial. *Vet. Dermatol.* **14**, 11–22 (2003). 12603681 10.1046/j.1365-3164.2003.00318.x

[44] Vercelli, A., Raviri, G. & Cornegliani, L. The use of oral cyclosporin to treat feline dermatoses: a retrospective analysis of 23 cases. *Vet. Dermatol.* **17**, 201–206 (2006). 16674736 10.1111/j.1365-3164.2006.00514.x

[45] Wisselink, M. A. & Willemse, T. The efficacy of cyclosporine A in cats with presumed atopic dermatitis: a double blind, randomised prednisolone-controlled study. *Vet. J.* **180**, 55–59 (2009). 18294881 10.1016/j.tvjl.2007.11.018

[46] Speer, B. *Current Therapy in Avian Medicine and Surgery* (Elsevier Health Sciences, 2015).

[47] Sellon, D. C. et al. Infection of immunodeficient horses with *Sarcocystis neurona* does not result in neurologic disease. *Clin. Vaccine Immunol.* **11**, 1134–1139. 10.1128/cdli.11.6.1134-1139.2004 (2004). 10.1128/CDLI.11.6.1134-1139.2004 PMC524751 15539518

[48] Alper, G. Acute disseminated encephalomyelitis. *J. Child. Neurol.* **27**, 1408–1425. 10.1177/0883073812455104 (2012). 22914374 10.1177/0883073812455104

[49] Koibuchi, T. et al. Acute disseminated encephalomyelitis following *Plasmodium Vivax* malaria. *J. Infect. Chemother.* **9**, 254–256. 10.1007/s10156-003-0244-8 (2003). 14513395 10.1007/s10156-003-0244-8

[50] Dale, R. C. Acute disseminated encephalomyelitis. *Semin Pediatr. Infect. Dis.* **14**, 90–95. 10.1053/spid.2003.127225 (2003). 12881796 10.1053/spid.2003.127225

[51] Dubey, J. P. Development of immunity to sarcocystosis in dairy goats. *Am. J. Vet. Res.* **42**, 800–804 (1981). 6789727

[52] Dubey, J. P. Immunity to sarcocystosis: modification of intestinal coccidiosis, and disappearance of sarcocysts in dairy goats. *Vet. Parasitol.* **13**, 23–34 (1983). 6414153 10.1016/0304-4017(83)90017-1

[53] Erber, M. & Geisel, O. Untersuchungen Zur klinik und pathologie der *Sarcocystis-suicanis*-Infektion beim Schwein. *Berl Munch. Tierarztl. Wochenschr.*. **92**, 197–202 (1979). 110311

[54] Fayer, R. & Dubey, J. P. Protective immunity against clinical sarcocystosis in cattle. *Vet. Parasitol.* **15**, 187–201 (1984). 6437053 10.1016/0304-4017(84)90071-2

[55] Gut, J. Infection of mice immunized with formolized cystozoites of *Sarcocystis dispersa* cerna, Kolarova et sulc, 1978. *Folia Parasitol.* **29**, 285–288 (1982). 6813204

[56] O’Donoghue, P. J., Rommel, M., Weber, M. & Weyreter, H. Attempted immunization of swine against acute sarcocystosis using cystozoite-derived vaccines. *Vet. Immunol. Immunopathol.* **8**, 83–92 (1985). 3919497 10.1016/0165-2427(85)90112-6

